# Genomic amplification patterns of human telomerase RNA gene and C-MYC in liquid-based cytological specimens used for the detection of high-grade cervical intraepithelial neoplasia

**DOI:** 10.1186/1746-1596-7-40

**Published:** 2012-04-13

**Authors:** Shaomin Chen, Ziyan Yang, Yun Zhang, Yunbo Qiao, Baoxia Cui, Youzhong Zhang, Beihua Kong

**Affiliations:** 1 Department of Obstetrics and Gynecology, Qilu Hospital, Shandong University, Jinan, Shandong 250012, People's Republic of China

**Keywords:** Uterine cervical neoplasia, Oncogenes, Fluorescence in situ hybridization, Telomerase RNA gene, C-MYC, Human papillomavirus

## Abstract

**Background:**

The amplification of oncogenes initiated by high-risk human papillomavirus (HPV) infection is an early event in cervical carcinogenesis and can be used for cervical lesion diagnosis. We measured the genomic amplification rates and the patterns of human telomerase RNA gene (TERC) and C-MYC in the liquid-based cytological specimens to evaluate the diagnostic characteristics for the detection of high-grade cervical lesions.

**Methods:**

Two hundred and forty-three residual cytological specimens were obtained from outpatients aged 25 to 64 years at Qilu Hospital, Shandong University. The specimens were evaluated by fluorescence in situ hybridization (FISH) using chromosome probes to TERC (3q26) and C-MYC (8q24). All of the patients underwent colposcopic examination and histological evaluation. A Chi-square test was used for categorical data analysis.

**Results:**

In the normal, cervical intraepithelial neoplasia grade 1 (CIN1), grade 2 (CIN2), grade 3 (CIN3) and squamous cervical cancer (SCC) cases, the TERC positive rates were 9.2%, 17.2%, 76.2%, 100.0% and 100.0%, respectively; the C-MYC positive rates were 20.7%, 31.0%, 71.4%, 81.8% and 100.0%, respectively. The TERC and C-MYC positive rates were higher in the CIN2+ (CIN2, CIN3 and SCC) cases than in the normal and CIN1 cases (*p *< 0.01). Compared with cytological analysis, the TERC test showed higher sensitivity (90.0% vs. 84.0%) and higher specificity (89.6% vs. 64.3%). The C-MYC test showed lower sensitivity (80.0% vs. 84.0%) and higher specificity (77.7% vs. 64.3%). Using a cut-off value of 5% or more aberrant cells, the TERC test showed the highest combination of sensitivity and specificity. The CIN2+ group showed more high-level TERC gene copy number (GCN) cells than did the normal/CIN1 group (*p *< 0.05). For C-MYC, no significant difference between the two histological categories was detected (*p *> 0.05).

**Conclusions:**

The TERC test is highly sensitive and is therefore suitable for cervical cancer screening. The C-MYC test is not suitable for cancer screening because of its lower sensitivity. The amplification patterns of TERC become more diverse and complex as the severity of cervical diseases increases, whereas for C-MYC, the amplification patterns are similar between the normal/CIN1 and CIN2+ groups.

**Virtual slides:**

The virtual slide(s) for this article can be found here: http://www.diagnosticpathology.diagnomx.eu/vs/1308004512669913.

## Background

Cervical cancer is widely recognized to be caused primarily by persistent infection with high-risk human papillomavirus (HPV). The integration of the HPV genome into the host genome results in the constitutive expression of the oncoproteins E6 and E7, which combine with the tumor suppressor genes P53 or RB to disrupt cell cycle regulation and initiate the crucial step of tumorigenesis [1-3]. HPV infection is necessary but not sufficient for cervical carcinogenesis. HPV infection is common and, in most cases, is self-limiting and can be eradicated; only a minority of the cases progress to cervical precancerous lesions. The contrast between the high rate of HPV infection and the low rate of associated cervical cancer morbidity suggests that additional genetic events are necessary for the malignant progression of cervical lesions [4]. The amplification of oncogenes is commonly observed in cervical precancerous lesions according to the results of comparative genomic hybridization (CGH) studies [5,6]. In contrast to chromosomal instability, oncogene amplification can occur even in an otherwise chromosomally stable cell and is a fairly early event in cervical carcinogenesis.

For cervical cancer screening, a single liquid-based cytological examination is relatively insensitive, has poor repeatability and often gives equivocal results. Used as a complementary procedure, the Hybrid Capture 2 (HC2) HPV DNA test is characterized by extremely high sensitivity but relatively low specificity. In clinical practice, high-grade lesions require immediate surgical treatment, whereas low-grade lesions may be closely monitored at defined intervals. This situation has prompted efforts to discover other biomarkers with the potential for high specificity as well as high sensitivity for the detection of high-grade lesions and cervical cancers. The change of a biomarker must be an early event in the process of cervical carcinogenesis. Oncogenes that are frequently amplified in precancerous lesions should be taken into consideration.

The pattern of chromosomal imbalances in cervical cancer is conserved. We reviewed relevant literature and found that TERC (3q26) and C-MYC (8q24) are the two most frequently observed amplified oncogenes in cervical precancerous lesions according to the results of CGH studies [5,6]. TERC, the RNA component of human telomerase, is the most frequently observed amplified oncogene and is presumed to play a central role in cervical carcinogenesis [5-8]. TERC amplification was observed in 35% of cervical intraepithelial neoplasia grade 3 (CIN3) cases and in 72% of invasive cancers [6]. The C-MYC (8q24) locus is the most commonly observed integration site of the HPV genome [9-13]. C-MYC amplification is frequently detected in precancerous cervical lesions with HPV infection. C-MYC may promote the immortality of the precancerous cells by directly activating the transcription of telomerase reverse transcriptase (TERT) [14]. In a study by Policht et al. (2010) [15], C-MYC positivity rates in normal subjects, CIN1, CIN2, CIN3 and cancer patients were 5%, 26%, 96%, 95% and 100%, respectively, and increased in association with the severity of the histological diagnosis. These biomarkers have great potential for use as tools in routine cervical diagnostics, and researchers have focused on the choice of test method and cut-off value.

The aims of the present study were to explore the distribution of the oncogene amplification patterns of TERC and C-MYC among women undergoing liquid-based cytological examination during population-based screening, and to compare the fluorescence in situ hybridization (FISH) results with the underlying histology of the cytological specimens at various cut-off values to assess the diagnostic characteristics of these biomarkers in the diagnosis of CIN2+. Interphase FISH for TERC and C-MYC was performed on residual liquid-based cytological specimens. The ratios of aberrant cell count/observed cell count and oncogene amplification patterns were recorded. All cases underwent colposcopic examination and were histologically confirmed.

## Materials and methods

### Specimens

**Figure 1:**
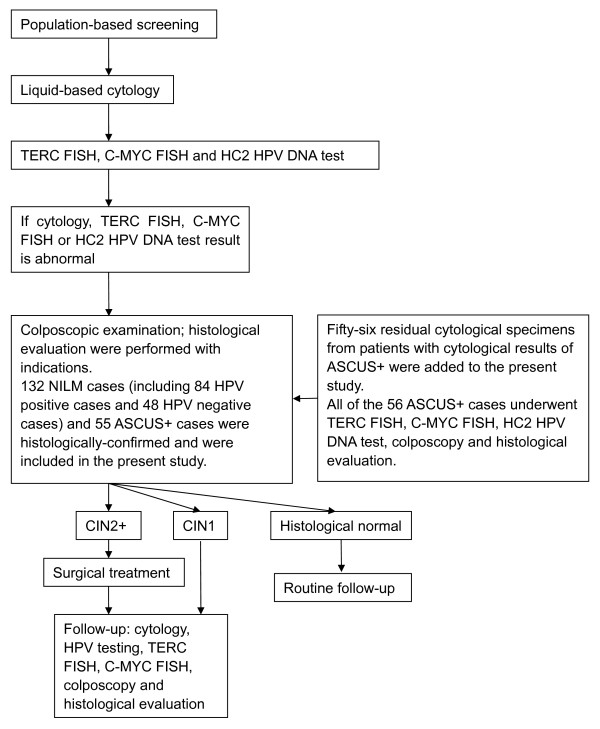
**Flow chart of the study design**.

Residual PreservCyt (Cytyc) cytological specimens from 243 outpatients (aged 25 to 64 years) seen at Qilu Hospital, Shandong University (Jinan, Shandong, China) between August 2010 and October 2011 were obtained. One hundred and thirty-two cases that were negative for intraepithelial lesion or malignancy (NILM), 50 cases of atypical squamous cells of undetermined significance (ASCUS), 21 cases of low-grade squamous intraepithelial lesion (LSIL), 14 cases of atypical squamous cells that cannot be excluded for high-grade squamous intraepithelial lesion (ASC-H), 23 cases of high-grade squamous intraepithelial lesion (HSIL) and 3 cases of squamous cell carcinoma (SCC) were included. All of the 132 NILM cases (including 84 HPV-positive cases and 48 HPV-negative cases) and 55 cytologically abnormal cases (ASCUS or worse; ASCUS+) were obtained from a large-scale opportunistic screening program between August 2010 and February 2011 (detailed screening program design see flow chart in Figure 1). To facilitate statistical analysis of the amplification patterns, another 56 ASCUS + cases were recruited from February 2011 to October 2011. Patients who were confirmed to have histological CIN and had undergone colposcopic examination or treatment for cervical lesions were excluded from the study. The study was approved by the ethics committee of Qilu Hospital, Shandong University and performed in accordance with the ethical standards. All of the specimens had been used previously for clinical purposes, were no longer clinically necessary and were used for the present study with the informed consent of the patients. The results of liquid-based cytological analysis were classified according to the 2001 Bethesda System [16]. In cases of controversial results, a consensus on the result was reached by two cytopathologists. Colposcopic examinations and histological evaluations were performed within 4 months for all cases, including normal and dysplasia cases. The pathologists who evaluated the biopsy specimens were unaware of the cytological results, and any discrepancies were resolved by two pathologists. The residual cytological specimens were stored at 4°C for the FISH test. Each specimen was incubated with collagen B and deionized water, fixed with methanol-acetic acid, stored at -20°C and tested by FISH within 1 month.

### Cervical cancer-specific FISH probe panel

The two most frequently amplified oncogenes detected in cervical precancerous lesions in previous CGH studies, TERC and C-MYC, were evaluated by FISH in the present study for their amplification patterns in cytological specimens. The panel consisted of two groups: (i) dual-color FISH probes for TERC (labeled with spectrum red) and centromere 3 (CEP3, labeled with green; used to evaluate the amplification of arm vs. polyploidy); (ii) C-MYC (labeled with red). All of the probes were provided by GP Medical Technologies (Beijing, China). Five to ten milliliters of cell preserved liquid from each specimen was centrifuged to collect cells. The collected cells were incubated with collagen B at 37°C for 20 min and then with deionized water at 37°C for 30 min. The treated cells were twice fixed in methanol-acetic acid (3:1) for 10 min. The cell suspensions were then transferred to microscope slides by a dropper; one drop was used for the TERC test, and another was used for the C-MYC test. The slides were dried at 56°C for 30 min, and the shape and number of cervical epithelial cells were observed under a light microscope with a 10× objective by limiting the light through the diaphragm. Cell numbers of approximately 200 to 800 were used because smaller cell numbers are not sufficient for evaluation and too many cells often clump together. The slides were washed twice in 2 × SSC for 5 min, treated with 0.1 M HCl for 10 min, digested with 0.02 mg/ml pepsin/0.01 M HCl at 37°C for 10 min, fixed in 2.5% formaldehyde/PBS for 10 min, dehydrated in an ethanol series and air dried. The slides and probes were denatured simultaneously at 75°C for 5 min followed by hybridization in a wet box at 42°C for 16 h. The cover slips were removed, and the slides were washed in 0.3% NP-40/0.4 × SSC at 67°C for 2 min, in 0.1% NP-40/2 × SSC for 30 s and in 70% ethanol for 3 min. The slides were counterstained with 4,6-diamidino-2-phenylindole (DAPI) for 10-20 min, and then screened with a 40× objective to observe the hybridization quality. Slides were considered disqualified if more than 25% of the cells were insufficiently hybridized. In this situation, we transferred suspended cells onto the slides again and repeated the hybridization procedure.

### Signal enumeration

FISH images were acquired using an Olympus BX51 fluorescence microscope (Tokyo, Japan) that was connected to a ProgRes Mfcool JENOPTIK camera (Jena, Germany). Multilayer images were then acquired for all of the probes in the probe panel using VideoTesT-FISH 2.0 software. The slides were screened with a 100× objective through a DAPI filter to determine the cell areas by starting at one corner of the slide and advancing from one field of view to the next in an orderly manner. We enumerated TERC signals through a red filter and CEP3 signals through a green filter at one slide and C-MYC signals through a red filter on another slide. Several continuous visions were evaluated and the signals from all cells in the visions were enumerated. At least 100 nuclei for each probe were evaluated and the proportion of abnormal cells was calculated [17,18]. Next, we rapidly screened the residual areas. In most cases the ratio of aberrant cells to observed cells remained stable. When small populations of aberrant cells within a large cell population were detected, focal precancerous lesions could not be ruled out. In these cases, we enumerated several other visions including the one with the aberrant cell crowd.

**Figure 2:**
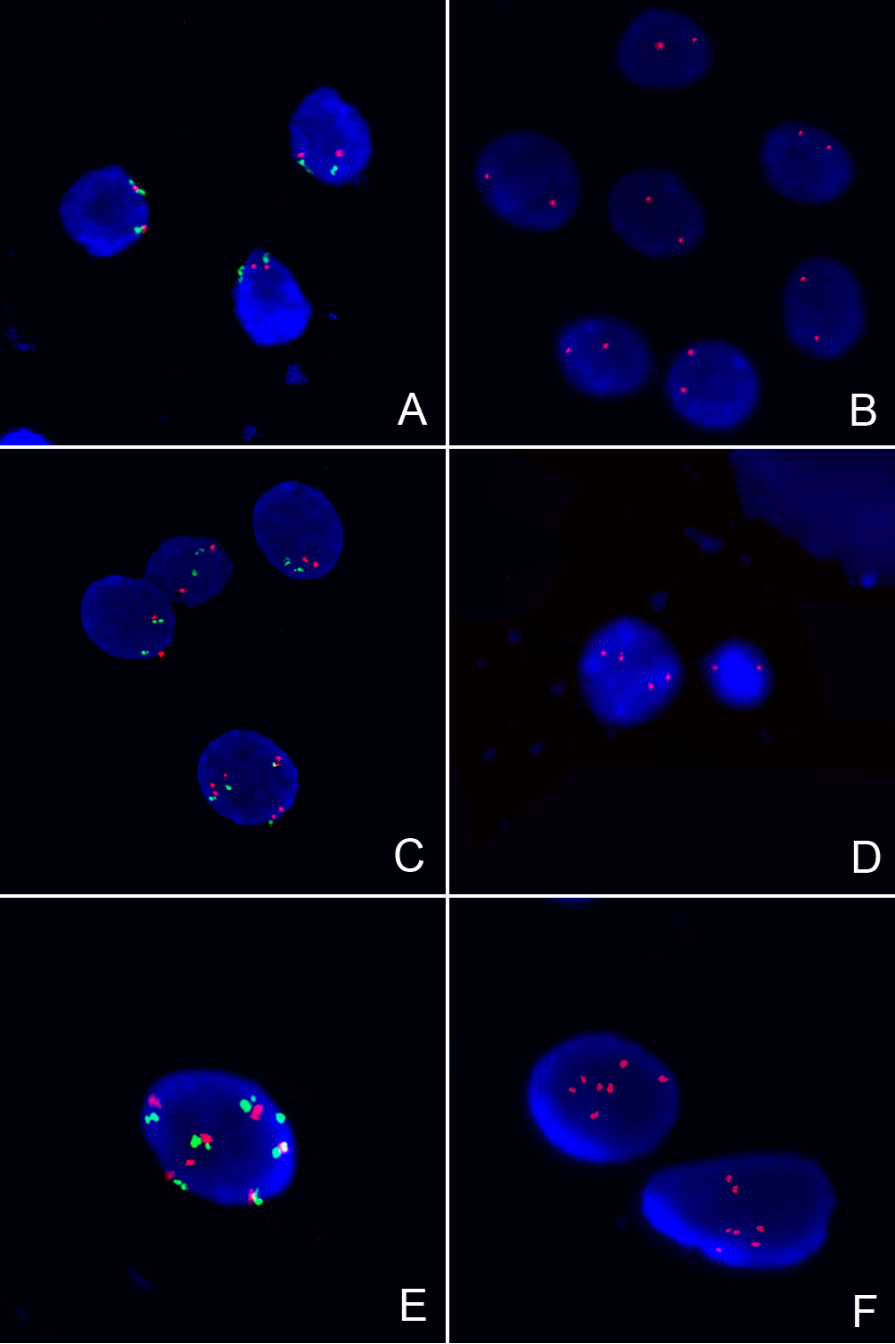
**Representative images of probe set TERC -CEP3 and C-MYC signals observed in cervical epithelial cells**. A: Normal TERC-CEP3 signal pattern of 2-2 (patterns are described in the order TERC-CEP3). B: Normal C-MYC signal pattern with 2 signals in each nucleus. C: A 7-4 pattern cell of TERC-CEP3 hybridization is shown at the bottom. The cytological diagnosis was LSIL, whereas the histological diagnosis was CIN2. D: A 4-signal cell of C-MYC hybridization is shown at the left. The cytological diagnosis was HSIL, but the histological diagnosis was CIN1. E: A 7-7 signal pattern cell from a HSIL patient. The histological diagnosis was CIN3. F: Two 7-signal cells that lay next to each other in an HSIL patient. The histological diagnosis was CIN3.

Normal diploid cells contain 2 signals of each probe in a nucleus, and a 2:2 signal ratio of TERC to CEP3 indicates a normal signal pattern. A cell was considered to be chromosomally abnormal if either TERC or C-MYC probe showed 3 or more signals per cell (Figure 2). When two signals lay next to each other, they were considered to be a doublet if there was almost no space between them and each signal was smaller than normal. If there was apparent space between the two signals and they had the same size as normal signals, they were enumerated as two separate signals. A consensus diagnosis was made by two pathologists in cases of controversial enumerations regarding doublets. The pathologists who enumerated the signals were unaware of the cytological and histological results. The amplification patterns of TERC and C-MYC for each patient were recorded using a manual counter and then taken together. The results included ratios of abnormal cell count/observed cell count and hybridization patterns of aberrant cells, e.g., 3/100, 3-2 × 3.

### Statistical analysis

**Figure 3:**
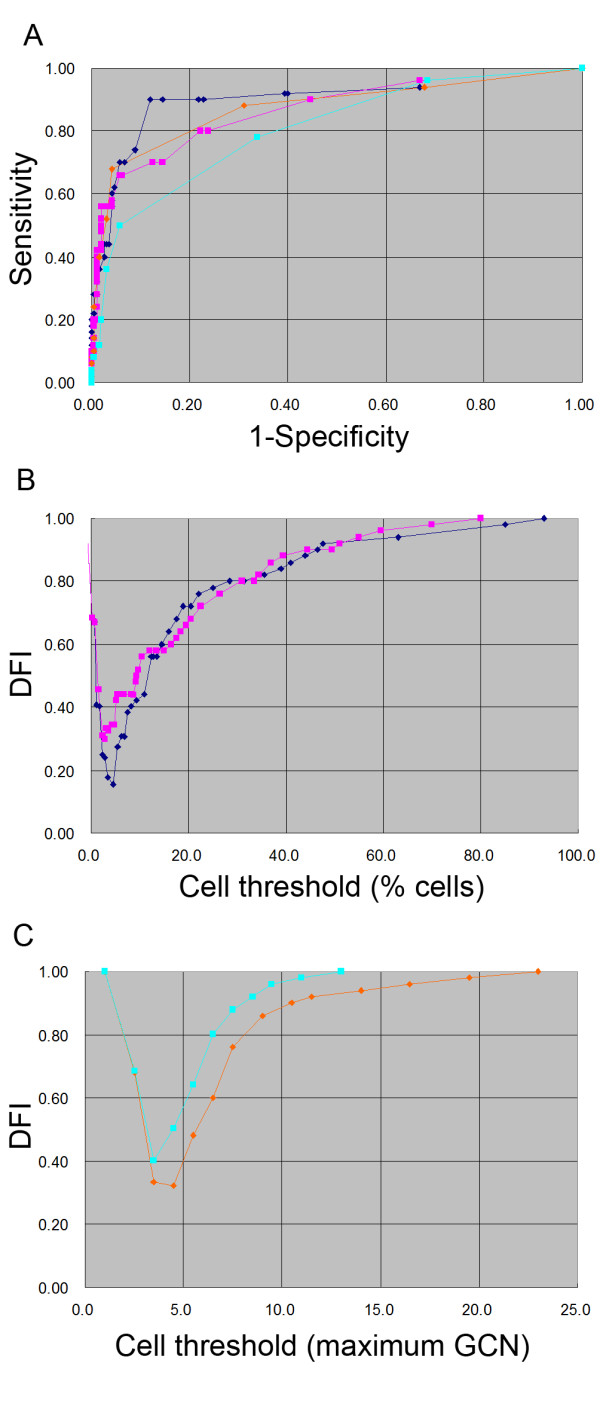
**ROC and DFI curves for aberrant cell percentage and maximum GCN of TERC and C-MYC gain for CIN2+ diagnosis**. A: Plots of sensitivity vs. 1-specificity at cut-off values of the percentage of aberrant cells and of maximum GCN for the TERC and C-MYC tests. B: Plots of DFI vs. cut-off values of the percentage of aberrant cells of the TERC and C-MYC tests. C: Plots of DFI vs. cut-off values at maximum GCN of the TERC and C-MYC tests. In A, B and C, blue diamonds indicate the results of TERC gain at cut-off values of aberrant cell percentage ranging from 0% to 100%; pink squares indicate the results of C-MYC gain at cut-off values of aberrant cell percentage ranging from 0% to 100%; orange diamonds indicate the results of TERC gain at cut-off values of GCN ranging from 1 to 23; slate green squares indicate the results of C-MYC gain at cut-off values of GCN ranging from 1 to 13.

A Chi-square test was used for 2 × 2 table analysis of the categorical data. A Z-test was used to assess whether the calculated rates of two groups were significantly different. Statistical significance was set at P value less than 0.05. Youden's index (Y = sensitivity + specificity - 1) was used to evaluate the combined sensitivity and specificity of the diagnostic methods. Receiver operator characteristic (ROC) and distance from ideal (DFI) curves were used to evaluate the optimal cut-off value at different aberrant cell percentages and gene copy number (GCN) for CIN2+ diagnosis. DFI was calculated as [(1 - sensitivity)^2 ^+ (1 - specificity)^2^]^1/2^. The curves closet to the ideal values of 100% sensitivity and 100% specificity (top left corner of ROC graph and bottom margin of DFI graph, Figure 3) provide the best combination of sensitivity and specificity, giving the equal weight to each.

## Results

### Cut-off values from various cell percentages and GCNs of the TERC test and the C-MYC test

ROC curves for various cell percentages and GCNs for CIN2+ diagnosis were produced using the data from all 243 cases (Figure 3A), and the areas under the curves (AUC) were calculated. The optimal cut-off value was ≥ 5% aberrant cells of TERC among the cut-off values of cell percentages and GCNs of TERC and C-MYC. Using this cut-off value, the TERC test showed 90.0% sensitivity and an ideal combination of sensitivity and specificity (Youden's index = 79.6%) in CIN2+ diagnosis. DFI curves for different cell percentages and for different GCNs were produced separately (Figure 3B, C). For the TERC test, the cut-off value of ≥ 5% TERC gain cells (AUC = 0.9, DFI = 0.2) showed a higher combined sensitivity and specificity than the cut-off value of TERC GCN ≥ 5 (AUC = 0.8, DFI = 0.3). For the C-MYC test, the cut-off value of ≥ 3% C-MYC gain cells (AUC = 0.8, DFI = 0.3) showed a higher combined sensitivity and specificity than the cut-off value of C-MYC GCN ≥ 4 (AUC = 0.7, DFI = 0.4). We chose ≥ 5% TERC gain cells as the cut-off value of the TERC test and ≥ 3% C-MYC gain cells as the cut-off value of the C-MYC test for statistical analysis.

### The association between TERC and C-MYC amplification rates and cytopathological and histopathological evaluations

The number of cases with TERC and C-MYC amplifications according to cytological diagnoses is shown in Table 1. In the cytological diagnoses of NILM, ASCUS, LSIL, ASC-H, HSIL and SCC, the TERC positive rates were 8.3%, 20.0%, 52.4%, 64.3%, 91.3% and 100.0%, respectively, and the C-MYC positive rates were 22.0%, 26.0%, 57.1%, 42.9%, 87.0% and 100.0%, respectively. Significant differences were detected among normal (NILM), low-grade lesions (ASC/LSIL) and high-grade lesions (HSIL/SCC) for both TERC and C-MYC (*p *< 0.01). There were also significant differences between LSIL or lower and HSIL or higher for both TERC and C-MYC (*p *< 0.01). The number of cases with TERC and C-MYC amplifications with different histological results is shown in Table 2. For the histological diagnoses of normal, CIN1, CIN2, CIN3 and SCC, the TERC positive rates were 9.2%, 17.2%, 76.2%, 100.0% and 100.0%, respectively, and the C-MYC positive rates were 20.7%, 31.0%, 71.4%, 81.8% and 100.0%, respectively. The TERC and C-MYC positive rates were similar between normal and CIN1 but higher in CIN2+ than in CIN1 (*p *< 0.01). Significant differences were also observed between CIN1/lower and CIN2/higher (*p *< 0.01).

**Table 1:** TERC and C-MYC positivity rates for various cytological diagnoses

Cytological diagnosis	**Case no**.	TERC positive			C-MYC positive		
		
		**Case no**.	%	P	**Case no**.	%	P
NILM	132	11	8.3		29	22.0	
ASC-US	50	10	20.0		13	26.0	
LSIL	21	11	52.4		12	57.1	
ASC-H	14	9	64.3		6	42.9	
HSIL	23	21	91.3		20	87.0	
SCC	3	3	100.0		3	100.0	
NILM vs. ASC/LSIL				< 0.01*			< 0.01*
ASC/LSIL vs. HSIL/SCC				< 0.01*			< 0.01*
LSIL/lower vs. HSIL/higher				< 0.01*			< 0.01*

† ASC includes ASC-US and ASC-H

*P < 0.05: statistically significant difference between 2 categories

**Table 2:** TERC and C-MYC positivity rates for various histological diagnoses

Histological diagnosis	**Case no**.	TERC positive			C-MYC positive		
		
		**Case no**.	%	P	**Case no**.	%	P
Normal	164	15	9.2		34	20.7	
CIN1†	29	5	17.2		9	31.0	
CIN2	21	16	76.2		15	71.4	
CIN3	22	22	100.0		18	81.8	
SCC	7	7	100.0		7	100.0	
Normal vs. CIN1		> 0.05			> 0.05		
CIN1 vs. CIN2+		< 0.01*			< 0.01*		
Normal/CIN1 vs. CIN2+		< 0.01*			< 0.01*		

† CIN1 includes lesions previously classified as mild dysplasia, koilocytotic atypia, koilocytosis and flat condyloma

* *P *< 0.05: statistically significant difference between 2 categories

The association between FISH test positivity and cytological/histological diagnosis is shown in Table 3. Of the 8 NILM cases with underlying CIN2+, 2 TERC+/CMYC + cases, 1 TERC-/CMYC + case, 1 TERC+/CMYC- case and 4 TERC-/CMYC- cases were observed. For the 2 HSIL cases with normal histological results, both TERC and C-MYC tests were negative. For the 1 HSIL case with underlying CIN1, the cell percentages for the TERC test and the C-MYC test were 15% and 12%, respectively, but the amplification was at a low level (3-4 copies for the TERC test and the C-MYC test).

**Table 3:** Cervical test positivity rates for various clinical groups

Cytological/histological diagnosis	TERC positive cases [% (positive/total)]	C-MYC positive cases [% (positive/total)]
NILM-negative	7.3 (8/109)	19.3 (21/109)
NILM-CIN1	0.0 (0/15)	33.3 (5/15)
NILM-CIN2+	37.5 (3/8)	37.5 (3/8)
ASCUS-negative	12.8 (5/39)	23.1 (9/39)
ASCUS-CIN1	14.3 (1/7)	14.3 (1/7)
ASCUS-CIN2+	100.0 (4/4)	75.0 (3/4)
LSIL-negative	18.2 (2/11)	36.4 (4/11)
LSIL-CIN1	75.0 (3/4)	50.0 (2/4)
LSIL-CIN2+	100.0 (6/6)	100.0 (6/6)
ASC-H-negative	0.0 (0/3)	0.0 (0/3)
ASC-H -CIN1	0.0 (0/2)	0.0 (0/2)
ASC-H -CIN2+	100.0 (9/9)	66.7 (6/9)
HSIL-negative	0.0 (0/2)	0.0 (0/2)
HSIL-CIN1	100.0 (1/1)	100.0 (1/1)
HSIL-CIN2+	100.0 (20/20)	95.0 (19/20)
SCC-CIN2+	100.0 (3/3)	100.0 (3/3)

Of the 243 total cases, 48 TERC+/C-MYC + cases were detected, and 81.3% (39 of 48) of these cases showed histologically confirmed CIN2+. Of the 17 TERC+/C-MYC- cases, 35.3% (6 of 17) showed CIN2+. Of the 35 TERC-/C-MYC + cases, 1 showed CIN2+. Of the 143 TERC-/C-MYC-cases, 4 (2.8%) CIN2+ cases were confirmed by histological evaluation. A correlation between the amplifications of TERC and C-MYC was observed, with a correlation index of 0.506 (*p *< 0.01).

### The association between TERC and C-MYC amplification patterns and histological grades

We evaluated amplification patterns using the data of TERC and C-MYC positive cases (Table 4). From the TERC and C-MYC statistics, we found that the percentage of abnormal nuclei was significantly higher in the CIN2+ group than in the normal/CIN1 group (21.1 vs. 10.2 for TERC, *p *< 0.05; 20.9 vs. 6.2 for C-MYC, *p *< 0.05). The most frequently observed TERC abnormal GCN was 3-6 (83.2% for the CIN2+ group; 97.7% for the normal/CIN1 group). The CIN2+ lesions showed more GCN ≥ 7 cells than did the normal/CIN1 lesions (16.8% vs. 2.3%, *p *< 0.05). Cells with a TERC: CEP3 ratio of 1 were observed in 26.9% of the CIN2+ lesions and 33.8% of the normal/CIN1 lesions. The percentage of cells with a TERC: CEP3 ratio greater than 1 was higher in CIN2+ lesions than in normal/CIN1 lesions (69.1% vs. 61.0%, *p *< 0.05). For C-MYC, the percentage of cells with GCN = 3-6 was 97.0% for CIN2+ lesions and 98.3% for normal/CIN1 lesions. The percentage of cells with GCN ≥ 7 was 3.0% for CIN2+ lesions and 1.7% for normal/CIN1 lesions, and there was no significant difference between these two histological categories (*p *> 0.05).

**Table 4:** Proportions of TERC/C-MYC amplification types in cervical disorders

	TERC positive		C-MYC positive	
**Histological diagnosis**	**CIN2+**	**Normal/CIN1**	**CIN2+**	**Normal/CIN1**

Case no.	45	20	40	43
Abnormal nuclei (no.)	963	213	854	293
Abnormal nuclei (%)	21.1	10.2	20.9	6.2
Distribution of GCN (%)				
3 copies	27.7	55.9	48.0	49.5
4 copies	31.9	25.4	35.5	39.9
5 copies	16.5	10.8	9.6	7.2
6 copies	7.1	5.6	3.9	1.7
≥ 7 copies	16.8	2.3	3.0	1.7
Distribution of TERC: CEP3 ratios (%)				
< 1	4.0	5.2		
= 1	26.9	33.8		
> 1	69.1	61.0		

### Diagnostic performances of the TERC test and the C-MYC test for the detection of CIN2+

To assess the performance of the TERC test and the C-MYC test for evaluating high-grade cervical lesions, we calculated the sensitivity, specificity, PPV, NPV, accuracy and Youden's index of cytological analysis and FISH test for the diagnosis of CIN2+ lesions (Table 5). The diagnostic characteristics for a combined TERC and C-MYC test (scenario 1: both markers amplified = positive test; scenario 2: one of the markers amplified = positive test) are also included. We chose ASCUS+, which showed 84.0% sensitivity and 64.3% specificity, as the cut-off for cytological analysis. The diagnostic characteristics of the TERC and C-MYC tests used separately or in combination were calculated. Regarding the cut-off values, TERC + determined as ≥ 5% aberrant TERC cells and C-MYC + determined as ≥ 3% aberrant C-MYC cells.

**Table 5:** Comparison of cytological analysis and the FISH test for CIN2+ diagnosis

Test method	Cytological analysis	FISH test			
**Cut-off value†**	**ASCUS+**	**TERC+**	**C-MYC+**	**TERC + and C-MYC+**	**TERC + or C-MYC+**

Sensitivity, % (95%CI)	84.0 (73.8-94.2)	90.0(81.7-98.3)	80.0 (68.9-91.2)	78.0 (66.5-89.5)	92.0 (84.5-99.5)
Specificity, % (95%CI)	64.3(57.5-71.0)	89.6(85.3-93.9)	77.7 (71.8-83.6)	95.3 (92.3-98.3)	72.0 (65.7-78.3)
PPV, % (95%CI)	37.8(28.8-46.9)	69.2(58.0-80.5)	48.2 (37.5-59.0)	81.3 (70.3-92.3)	46.0 (36.2-55.8)
NPV, % (95%CI)	93.9(89.9-98.0)	97.2(94.8-99.6)	93.8 (90.1-97.5)	94.4 (91.2-97.6)	97.2 (94.5-99.9)
Accuracy, % (95%CI)	68.3(62.5-74.2)	89.7(85.9-93.5)	78.2 (73.0-83.4)	91.8 (88.4-95.3)	76.1 (70.7-81.5)
Youden's index, % (95%CI)	48.3(31.3-65.2)	79.6(67.0-92.3)	57.7 (40.7-74.8)	73.3 (58.8-87.5)	64.0 (50.2-77.8)

† Cut-off value: ASCUS + determined as ASCUS or higher. TERC + determined as ≥ 5% aberrant TERC cells among all of the cells observed. C-MYC + determined as ≥ 3% aberrant C-MYC cells among all of the cells observed

In comparison with cytological analysis, the TERC test showed higher sensitivity (90.0% vs. 84.0%) and specificity (89.6% vs. 64.3%). The accuracy and Youden's index of the TERC test were higher than those of cytological analysis (89.7% vs. 68.3%, 79.6% vs. 48.3%, respectively). In comparison with cytological analysis, the C-MYC test showed lower sensitivity (80.0% vs. 84.0%) but higher specificity (77.7% vs. 64.3%). The accuracy and Youden's index of the C-MYC test were higher than those of cytological analysis (78.2% vs. 68.3%, 57.7% vs. 48.3%, respectively). In comparison with the TERC test, the C-MYC test showed lower sensitivity (80.0% vs. 90.0%) and specificity (77.7% vs. 89.6%). If we combined the C-MYC and TERC tests and considered one of the markers amplified to be positive, the sensitivity increased from 90.0% to 92.0%, the specificity decreased from 89.6% to 72.0%, the accuracy decreased from 89.7% to 76.1% and Youden's index decreased from 79.6% to 64.0%. If we considered both of the markers amplified to be positive, the sensitivity decreased from 90.0% to 78.0%, the specificity increased from 89.6% to 95.3%, the accuracy increased slightly from 89.7% to 91.8% and Youden's index decreased from 79.6% to 73.3%.

## Discussion

Although liquid-based cytology is the procedure most often used for cervical cancer screening, it has limitations related to subjectivity and relative insensitivity. The high-risk HC2 HPV DNA test is sometimes performed because of its advantages of high sensitivity and NPV. However, most women infected with HPV will eliminate the virus within 1-2 years, and only a very small percentage of them will progress to high-grade diseases. The HPV DNA test is therefore a diagnostic method with high sensitivity but low specificity, particularly for younger, sexually active women [19,20]. It is now accepted that the integration of high-risk HPV into the host genome is one of the major contributing factors to cervical carcinogenesis, and this phenomenon can not only be observed in high-grade lesions but also in a proportion of low-grade lesions [21,22]. The HPV DNA test is incapable of distinguishing HPV physical status (episomal vs. integrated) and is therefore not effective for identifying which patients with HPV infections are likely to have a CIN2+ lesion. According to the WHO guideline [23] and the 2006 consensus guidelines of the American Society for Colposcopy and Cervical Pathology (ASCCP) [24], the recommendation for CIN1 cases is to undergo follow-up examinations at defined intervals, whereas the recommendation for CIN2/3 cases is to undergo immediate treatment for the prevention of progression to carcinoma. In view of the limitations of the currently used screening methods, more accurate and reliable predictive biomarkers are needed to complement morphologically based differential diagnostic methods. For the differential diagnosis of low- vs. high-grade lesions, the change of the biomarker should ideally be an early event in cervical carcinogenesis that occurs in precancerous lesions.

Amplification of oncogenes is commonly observed in cervical precancerous lesion. It is a fairly early event in cervical carcinogenesis. Researchers have applied FISH probes to TERC and C-MYC, the two most frequently observed amplified oncogenes in cervical precancerous lesions as detected by CGH studies, for cytological specimen analysis. Heselmeyer et al. (2003) [25] applied a FISH probe set to cervical cytological specimens and found that the TERC gain cell counts and the maximum TERC copies was correlated with the severity of cervical lesions. After a follow-up of 1 to 3 years, the percentage of TERC amplified cases increased from 52% to 96% [26]. Sokolova et al. (2007) [27] applied a TERC-MYC-HPV probe-mix to Thinprep slides and found that LSIL/HSIL cytological specimens with underlying CIN2/3 showed positive FISH test results in more than 80% cases at a cut-off value of 4 or more double-positive cells (HPV and TERC/MYC aberrations). Andersson et al. (2009) [28] applied a TERC-MYC-HPV probe-mix to Thinprep slides by a similar procedure but using different enumeration methods and cut-off values. All cells on the slides were enumerated, including HPV-positive and HPV-negative cells. This procedure increased the sensitivity by including the HPV-negative cells with oncogene amplification. The cut-off value of 9 cells with more than two TERC copies in a whole slide scan excluded the effect of HPV and MYC, and the cut-off value of the TERC test appeared to be higher than the cut-off value of Sokolova et al., thereby increasing the specificity of the FISH test.

The procedures for specimen processing and signal enumeration in our study differed somewhat from those of Sokolova et al. (2007) and Andersson et al. (2009). In our study, the slides for enumeration were prepared with cell suspension drops from residual Thinprep PreservCyt (Cytyc) cytological specimens. The cells were pre-treated with collagen B and deionized water and were thereby dispersed and enlarged. Because the cells were evenly mixed, the possible selection bias of enumeration vision was limited. The TERC probe and C-MYC probe were applied separately on two slides, in contrast to the two studies above. Regarding the comparison of enumeration methods, Sokolova et al. (2007) analyzed the entire surface area of each slide in most cases. However, when there was a large number of HPV-positive cells per slide, the first 100 HPV-positive cells were analyzed and the number of HPV-positive cells on a whole slide was extrapolated from the percentage of surface area occupied by the cells. Andersson et al. (2009) enumerated the signals by screening and counting the entire slide visually; an average of 2320 nuclei per slide (range: 232 to 4996 nuclei) were counted. Enumerating cells on a whole slide might be time-consuming and tedious. To achieve rapid and accurate screening for clinical usage, we enumerated only the first 100 cells on each slide and then screened the whole slide. The time cost for signal enumeration of a slide is determined by the number of cells on a whole slide and the complexity of hybridization patterns. It usually takes 30 to 60 min to evaluate both the TERC and C-MYC signals for one patient.

The cut-off value we used for CIN2+ diagnosis is 5% or more aberrant TERC cells, which is higher than the cut-off values used in the two studies above and is also higher than the cut-off value of ≥ 2.5% cells with more than 2 TERC signals used by Heselmeyer et al. (2003) [25]. However, the cut-off value we used is consistent with that used in a multicenter study in China, and the mean TERC test cut-off value determined for all of the participating centers was 6.4 ± 2.3% [18]. Regional and ethnic differences were not ruled out for cut-off value differences. Using this TERC test cut-off value, our study showed specificity values similar to those of Andersson et al. (89.6% and 83.9%, respectively) and higher sensitivity (90.0% and 78.7%, respectively) for CIN2+ diagnosis. As possible cut-off value choices for the C-MYC test, the values of ≥ 3% and ≥ 5% C-MYC gain cells showed similar AUC (0.8) and DFI (0.3) values. We chose ≥ 3% C-MYC gain cells as the cut-off value because of its higher sensitivity. Using this cut-off value, the C-MYC test showed a sensitivity of 80.0% and a specificity of 77.7%. The sensitivity and specificity of C-MYC would be similar to those of Andersson et al. if one used a cut-off value of ≥ 5% aberrant C-MYC cells (66.0% vs. 66.0%, and 94.8% vs. 87.1%, respectively).

High-level amplifications have certain indication roles for advanced-grade precancerous lesions. Compared with other types of solid tumors, cervical cancer has a relatively low-level of amplification, usually at 3 to 6 copies, and a minority of the nuclei have a GCN > 20. Tu et al. [17] found primarily TERC amplification patterns of 3 to 4 copies in the normal/CIN1 group, whereas in the CIN2+ group, the percentage of amplification patterns of 5 copies and 6 copies were 10.2% and 54.6%, respectively. In our study, we detected a SCC case with a TERC GCN of 22 and a CIN2 case with a C-MYC GCN of 12. We found that the percentage of abnormal nuclei increased with the severity of disease for both TERC and C-MYC (*p *< 0.05). The CIN2+ group showed more high-level TERC amplifications (GCN ≥ 7) than did the normal/CIN1 group. However, the C-MYC amplification patterns are similar between the normal/CIN1 and CIN2+ lesions. Although GCN is an indicator for CIN2+ diagnosis, the combined sensitivity and specificity of GCN is lower than that of aberrant cell percentage for CIN2+ diagnosis.

TERC amplification patterns become more diverse as histological grades increases. The formation of isochromosome 3q is frequently observed in cervical carcinogenesis. In CGH studies, gain of 3q and loss of 3p were usually observed simultaneously in cervical cancer. In the study by Kirchhoff et al. (1999) [6], none of the 29 invasive cancers analyzed showed an entire extra chromosome 3. A CEP3 probe was therefore used to evaluate the relationship between TERC amplification and polyploidy. A TERC: CEP3 ratio > 1 suggested isochromosome formation in a cell. Cells with a TERC: CEP3 ratio > 1 accounted for 61.0% of the cells in the normal/CIN1 group and 69.1% of the cells in the CIN2+ group. A TERC: CEP3 ratio of 1 was observed in 33.8% of the cells in the normal/CIN1 group and 26.9% of the cells in the CIN2+ group. The TERC: CEP3 ratio appears to be higher in the CIN2+ group than in the normal/CIN1 group, and the formation of an isochromosome provides a reasonable explanation for this observation.

In comparison with cytological analysis, the TERC test is suitable for CIN2+ diagnosis because of its high sensitivity (90.0%) and its optimal combination of sensitivity and specificity (Youden's index = 79.6). The C-MYC test is not suitable for cancer screening because its sensitivity and specificity are lower than those of the TERC test (80.0% vs. 90.0% and 77.7% vs. 89.6%, respectively). When we combined the C-MYC and TERC tests and considered one of the markers amplified to be positive, the sensitivity increased slightly from 90.0% to 92.0%, while the specificity decreased greatly from 89.6% to 72.0%. The C-MYC test showed marginally increased sensitivity for screening but reduced specificity.

The specimens used in the present study were primarily obtained from a population-based screening program in which residual cervical liquid-based specimens were used for the FISH test of TERC and C-MYC amplification and for the HC2 HPV DNA test. Patients with positive results (cytological analysis, TERC test, C-MYC test or HC2 HPV DNA test) were recommended for colposcopic examinations. Colposcopy-directed biopsy and histological evaluation were performed if indicated. The correlations among oncogene amplification, HPV infection and cytological-histological results were analyzed, and the design of an optimal strategy for cervical cancer screening is discussed in a separate article (Shaomin Chen, Yun Zhang, Yunbo Qiao, et al., manuscript in preparation). To analyze the correlation between oncogene amplification patterns and cytological/histological diagnosis in the present study, we chose all histologically confirmed cases, including 132 cases of NILM (including 84 HPV-positive cases and 48 HPV-negative cases; the 48 HPV-negative cases included 1 TERC+/C-MYC + case, 2 TERC+/C-MYC- cases, 10 TERC-/C-MYC + cases and 35 TERC-/C-MYC- cases; the 35 TERC-/C-MYC- cases were selected as a control group) and 55 cases of ASCUS+. This choice accounts for the higher number of histologically normal cases than abnormal cases. For the facilitation of the statistical analysis of amplification patterns, another 56 ASCUS + cases were recruited from February 2011 to October 2011. Although the number of abnormal cases is still rather small, the aberrant cells observed are sufficiently numerous for data analysis and it is possible to reach preliminary conclusions regarding the oncogene amplification patterns in CIN2+ cases. We collected detailed data (including contact information) from all of the patients who underwent screening for long-term follow-up. It is recommended that patients with negative screening results undergo routine screening and that patients with positive screening results undergo cytological analysis, FISH test, HPV test, colposcopy and histological evaluation at defined intervals.

The FISH test is suitable for clinical testing because of its several advantages. It can be performed using residual cytological specimens without additional sampling. The FISH test is a cell-based evaluation technique and is therefore more sensitive than other methods such as PCR and microarray-based analyses. The interpretation of fluorescent signals is objective and repeatable and does not rely heavily on highly trained personnel. We were interest in possible reasons for the discrepancy between FISH test results and cytological or histological results. We reviewed the slides of the 4 NILM/TERC-/CMYC- cases with underlying CIN2+ by screening the whole slide and found that the aberrant cell percentages were below the cut-off values and that the amplification patterns were simple (3-4 copies). We also reviewed the colposcopical and histological images from these cases and found that most of them were focal CIN2+ cases; therefore, sampling omissions could not be ruled out.

The preliminary results of this study indicate that TERC amplification is a clinically applicable genetic approach for cervical lesion diagnosis because of its high sensitivity and optimal combination of sensitivity and specificity. The C-MYC test cannot be used for screening because of its low sensitivity and because it does not result in increased specificity when used in combination with the TERC test. Compared to the C-MYC test, the TERC test shows more high-level amplification copies and more diverse amplification patterns in high-grade lesions. However, the sensitivity of the TERC test is lower when using a cut-off value for the GCN than for the cell percentage. Further investigation of the possible application of GCN for prognosis of cervical neoplasia is needed.

## Abbreviations

FISH: Fluorescence in situ hybridization; TERC: Telomerase RNA gene; HPV: Human papillomavirus; HC2: Hybrid capture 2; NILM: Negative for intraepithelial lesion or malignancy; ASC-US: Atypical squamous cells of undetermined significance; LSIL: Low-grade squamous intraepithelial lesion; ASC-H: Atypical squamous cells that cannot be excluded for high-grade squamous intraepithelial lesion; HSIL: High-grade squamous intraepithelial lesion; SCC: Squamous cell carcinoma; CIN: Cervical intraepithelial neoplasia; PPV: Positive predictive value; NPV: Negative predictive value; GCN: Gene copy number.

## Competing interests

The authors declare that they have no competing interests.

## Authors' contributions

SC: study design, experimental studies, data analysis and manuscript preparation. ZY: clinical studies, data analysis and manuscript preparation. YZ: experimental studies and data analysis. YQ: experimental studies and manuscript review. BC: clinical studies and manuscript review. YZ: the guarantor of integrity of the entire study, study design, experimental studies, data analysis and manuscript preparation. BK: study design and manuscript review. All authors read and approved the final manuscript.
